# Multiscale genetic connectivity, kinship, and demographic history of the Antarctic soft coral *Alcyonium antarcticum* in the Western Antarctic Peninsula

**DOI:** 10.1038/s41598-026-47577-5

**Published:** 2026-04-26

**Authors:** Paulina Bruning, Leyla Cárdenas, Marie-Laure Guillemin, Ignacio Garrido, Josefina Jorquera, Philippe Archambault

**Affiliations:** 1 Department of Biology, Takuvik International Research Laboratory (CNRS – Université Laval – Sorbonne Université), Québec Ocean, Université Laval, Québec, QC Canada; 2 Centro FONDAP de Investigación en Dinámica de Ecosistemas Marinos de Altas Latitudes (IDEAL), Valdivia, Chile; 3 Laboratorio Costero Calfuco (LCC), Facultad de Ciencias, Universidad Austral de Chile, Valdivia, Chile; 4 Instituto de Ciencias Ambientales y Evolutivas, Facultad de Ciencias, Universidad Austral de Chile, Valdivia, Chile; 5 Núcleo Milenio MASH, Instituto de Ciencias Ambientales y Evolutivas, Facultad de Ciencias, Universidad Austral de Chile, Valdivia, Chile

**Keywords:** Ecology, Ecology, Evolution, Zoology

## Abstract

Rapid environmental change and a complex glacial history in the Western Antarctic Peninsula (WAP) have likely shaped the structure and persistence of Antarctic benthic species, yet connectivity remains poorly resolved for key habitat‑forming taxa. Understanding spatial genetic structure in these species is essential to predict their resilience and to inform conservation in this rapidly warming region. Here, we used genome‑wide SNPs to assess connectivity in the Antarctic soft coral *Alcyonium antarcticum* across multiple spatial scales along the WAP, sampling 90 individuals from three regions spanning the northern, central, and southern peninsula, with additional local and fine‑scale sampling within King George Island. Using DArTseq‑derived SNPs and a hierarchical sampling design, we analysed genetic diversity, population structure, and gene flow across large (hundreds of kilometers), local (kilometers), and fine (meters) spatial scales. At the large scale, three genetic clusters corresponding to Adelaide, Doumer, and King George islands indicated strong structure consistent with persistent historical and oceanographic barriers. At the local scale, significant differentiation, isolation by distance, and high self‑recruitment revealed weakly connected subpopulations, while at the fine scale the absence of clones indicated predominantly sexual reproduction with limited larval dispersal. Genetic diversity was lower than in temperate and tropical octocorals, consistent with glacial bottlenecks. Overall, *A. antarcticum* forms highly structured and weakly connected populations, highlighting the need to protect multiple demographically independent populations in the rapidly warming WAP.

**Supplementary Information:**

The online version contains supplementary material available at 10.1038/s41598-026-47577-5.

## Introduction

Population connectivity in benthic marine species is driven by the production, dispersal, settlement, and survival of larvae, and determines how spatially separated populations are demographically linked^1^. In contrast, genetic connectivity refers specifically to the exchange of genetic material among populations via the successful reproduction of migrants and captures the extent to which gene flow shapes evolutionary processes within subpopulations^2–4^.

In benthic marine species, genetic connectivity exhibits substantial variability and is shaped by both intrinsic biological traits and extrinsic environmental factors^5^. Intrinsic traits include larval duration, larval behavior, and life-history strategies^6^. Additionally, modes of development (direct versus indirect), influence dispersal potential. Beyond intrinsic biological traits, extrinsic factors—particularly oceanographic processes—play a fundamental role in determining population connectivity^7^. Moreover, historical demographic processes, including glacial contractions and subsequent post-glacial expansions, can leave long-lasting genetic signatures that may confound inferences of present-day gene flow^8^.

Given that genetic connectivity has direct implications for the resilience and sustainability of marine populations (e.g.,^9^), characterizing its spatial scales represents a key challenge for conservation. For example, the estimations of patterns of connectivity at distinct geographic scales is crucial for the design and management of Marine Protected Areas (MPAs), ensuring the preservation of vulnerable and economically valuable species^3,10,11^. At regional and large scales, rare long-distance dispersal processes, especially when they overcome the effect of strong geographic barriers and historical demographic events (i.e., contraction and expansion during glacial cycles) have been shown to drive genetic differentiation^12^. In contrast, connectivity at fine and local scales depend more on key biological traits and reproductive strategies, and local and mesoscale marine circulation processes such as eddies, upwelling plumes or tidal currents^13–16^, as well as recruitment dynamics and neighborhood interactions that can strongly affect local patterns of genetic structure^17^.

Our study focuses on Antarctic octocorals, using the family Alcyoniidae (Cnidaria: Octocorallia) as a representative example of one of the dominant suspension-feeding taxa in Antarctic benthic ecosystems^18,19^. Octocorals are sessile organisms that develop three-dimensional structures, increasing habitat complexity and providing refuges from predators and nursery areas for associated biodiversity^20–22^. For these reasons, they are considered ecosystem engineers^23^. However, their long lifespan and slow growth make them highly vulnerable to mechanical disturbance and the impacts of climate change^24,25^. Consequently, octocoral meadows have been recognized as Vulnerable Marine Ecosystems (VMEs) by the Food and Agriculture Organization (FAO)^26^, requiring protection from anthropogenic impacts and climate change^27^.

The octocoral *Alcyonium antarcticum* (Wright & Studer, 1889) is a lobate soft coral widely distributed across Antarctic and sub-Antarctic regions^28^, with populations occurring at depths ranging from 25 m to nearly 650 m^29^. Only one previous study has investigated the population genetic structure of *A. antarcticum* using nuclear (28 S) and mitochondrial (COI and MutS) markers^30^. This recent study, which analyzed samples collected from highly distant sites located in southern South America, the Falkland Islands, the Scotia Arc area and the Western Antarctic Peninsula (WAP), revealed the presence of cryptic species/highly divergent lineages. These cryptic species are distributed in allopatry, with *A. antarcticum* restricted to the WAP. Like other soft corals, *A. antarcticum* typically exhibit patchy distributions and can be locally abundant in sheltered habitats. Despite its importance as habitat forming species along the WAP, the biology and reproductive strategies of *A. antarcticum* remain unknown. Reproductive patterns among Antarctic octocorals are typically characterized by traits such as non-pelagic or lecithotrophic larval development, brooding, offspring protection, viviparity, slow embryonic development, advanced juvenile stages at hatching, and slow growth^31,32^. Genetic studies on octocorals from the Western Antarctic Peninsula (WAP) remain extremely limited^30,33,34^.

Given the patchy distribution of these suspension-feeding organisms in Antarctic waters (e.g.,^18,35,36^) and the increasing anthropogenic threats to these marine ecosystems, it is crucial to better understand the connectivity patterns and genetic diversity of *A. antarcticum.* Indeed, the Antarctic Peninsula is one of the fastest-warming regions on the planet^37,38^, and this rapid warming has profound ecological consequences. Rising seawater temperatures, increased iceberg scouring, ocean acidification, glacier meltwater runoff, sediment influx, and changes in sea-ice extent and ocean circulation^39,40^ represent stressors that negatively influence the reproductive success of benthic organisms^41^, directly affecting the functioning of marine ecosystems^42,43^. In this context, local populations of *A. antarcticum* could be highly reduced or even disappear, potentially affecting gene exchange at various geographical scales. Understanding actual patterns of genetic connectivity and diversity can help to assess the species’ ability to adapt and persist in this rapidly changing environment^44,45^.

This study provides the first genome-wide, multiscale assessment of connectivity and demographic history in the Antarctic soft coral *Alcyonium antarcticum* using a hierarchical sampling design and DArTseq-derived single nucleotide polymorphisms (SNPs). Our multiscale framework allows the investigation of distinct evolutionary and ecological processes depending on the spatial scale considered: (i) at the large scale (hundreds of kilometers between sampling sites along the Western Antarctic Peninsula), we aimed to identify patterns of long-distance dispersal, evaluate the role of geographic barriers, and assess the influence of historical events in shaping genetic differentiation among Antarctic regions; (ii) at the local scale (a few kilometers between sites within the same bay, in King George Island), we evaluated the presence of isolation by distance, kinship structure, and localized recruitment dynamics, providing insights into the species’ reproductive strategies and dispersal capacity; and, (iii) at the fine scale (within a single rocky reef, with colonies separated by only a few meters), we assessed the presence of clonal individuals and analyzed genetic structure at the neighborhood level. This scale is particularly relevant for *A. antarcticum*, as these octocorals typically form dense aggregations on rocky substrates, where short-distance larval dispersal and potential clonal propagation may shape fine-scale patterns of genetic diversity.

## Results

### SNP dataset summary

**Fig. 1:**
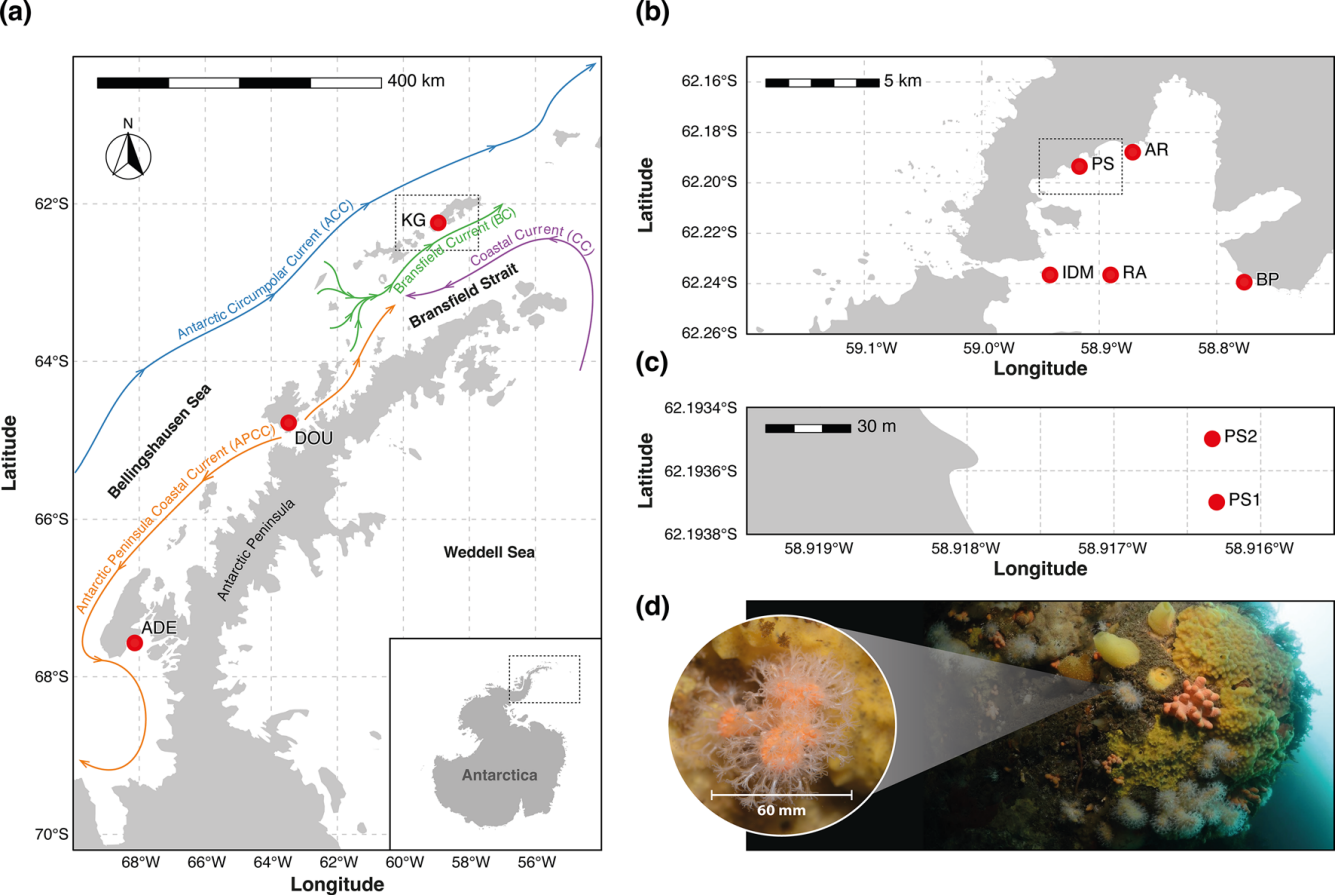
Map showing the study areas (red circles); at the large sampling scale of the Antarctic Peninsula (**a**), the local sampling scale of the Fildes Bay in King George Island (**b**), and the fine sampling scale in Punta Suffield (**c**). Typical habitat of *Alcyonium antarcticum* colonies used in this study, collected at approximately 20 m depth (**d**). Sites codes are: ADE (Adelaide Island), DOU (Doumer Island), KG (King George Island), BP (Barton Peninsula), AR (Artigas), PS (Punta Suffield), RA (Ras Tu), IDM (Islote Dos Morros), PS1 (Punta Suffield rock 1), and PS2 (Punta Suffield rock 2). More information about sites location is given in Table 1. Photo credit: Ignacio Garrido.

In total, 90 individuals were analysed. The large-scale dataset included samples from three regions—Adelaide Island (ADE), Doumer Island (DOU), and King George Island (KG)—while the local/fine-scale dataset comprised six sites within King George Island (Fig. 1). These datasets were used for downstream analyses following the hierarchical sampling design.

**Table 1:** Summary of genetic diversity estimates of *Alcyonium antarcticum* along the Antarctic Peninsula derived from 1,577 SNPs for the large scale data set and 2,219 SNPs in the local/fine and large data set. Missing data after filtering were low: 2.1% in the large-scale dataset and 1.4% in the local/fine-scale dataset (see Methods).

Scale	*N*	Site	Code	Lat. (S)	Long. (W)	H_o_	H_e_	F_is_
Large	10	King George Island^$^	KG	−62.24	−58.94	0.088 (0.158)	0.098 (0.161)	0.017*
	10	Doumer Island	DOU	−64.78	−63.47	0.073 (0.163)	0.076 (0.152)	0.089*
	11	Adelaide Island	ADE	−67.57	−68.14	0.121 (0.166)	0.247 (0.175)	0.536*
Local	10	Barton Peninsula	BP	−62.26	−58.78	0.088 (0.158)	0.098 (0.161)	0.017*
	9	Artigas	AR	−62.19	−58.87	0.088 (0.159)	0.098 (0.161)	0.148*
	10	Islote Dos Morros	IDM	−62.24	−58.94	0.098 (0.175)	0.098 (0.160)	0.053*
	10	Ras Tu	RA	−62.22	−58.89	0.095 (0.173)	0.099 (0.161)	0.084*
Fine	9	Punta Suffield 1	PS1	−62.19	−58.92	0.096 (0.168)	0.098 (0.161)	0.075*
	11	Punta Suffield 2	PS2	−62.19	−58.92	0.098 (0.176)	0.102 (0.164)	0.091*

*N* Number of individuals, *Ho* observed heterozygosity; *He* expected heterozygosity, *FIS* inbreeding coefficient. Values in parentheses represent standard deviations across loci. Asterisks indicate significant deviation from Hardy–Weinberg equilibrium (*p* < 0.05). $ KG and BP represent the same individuals from Barton Peninsula analysed under two independent scale-specific SNP datasets (large-scale and local/fine-scale).

DArTseq genotyping yielded 31,444 raw SNPs. After quality filtering, 1,574 neutral SNPs were retained for the large-scale dataset and 2,219 for the local/fine-scale dataset. Missing data were low in both datasets. The large-scale dataset (1,574 SNPs; *n* = 31 individuals) showed 2.1% missing genotypes on average (max per SNP 9.7%; max per individual 5.3%), whereas the local/fine-scale dataset (2,219 SNPs; *n* = 59 individuals) showed 1.4% missing genotypes on average (max per SNP 8.5%; max per individual 4.6%).

### Large geographic scale: genetic patterns along the Western Antarctic Peninsula

At the large scale, observed heterozygosity ranged from 0.073 (DOU) to 0.121 (ADE), and expected heterozygosity from 0.076 to 0.247 (Table 1). ADE showed higher diversity than both DOU and KG, with differences supported by non-overlapping 95% bootstrap confidence intervals (Table 1), while DOU and KG did not differ. Inbreeding coefficients ranged from 0.017 (KG) to 0.536 (ADE), and all were statistically significant (Table 1).

**Fig. 2:**
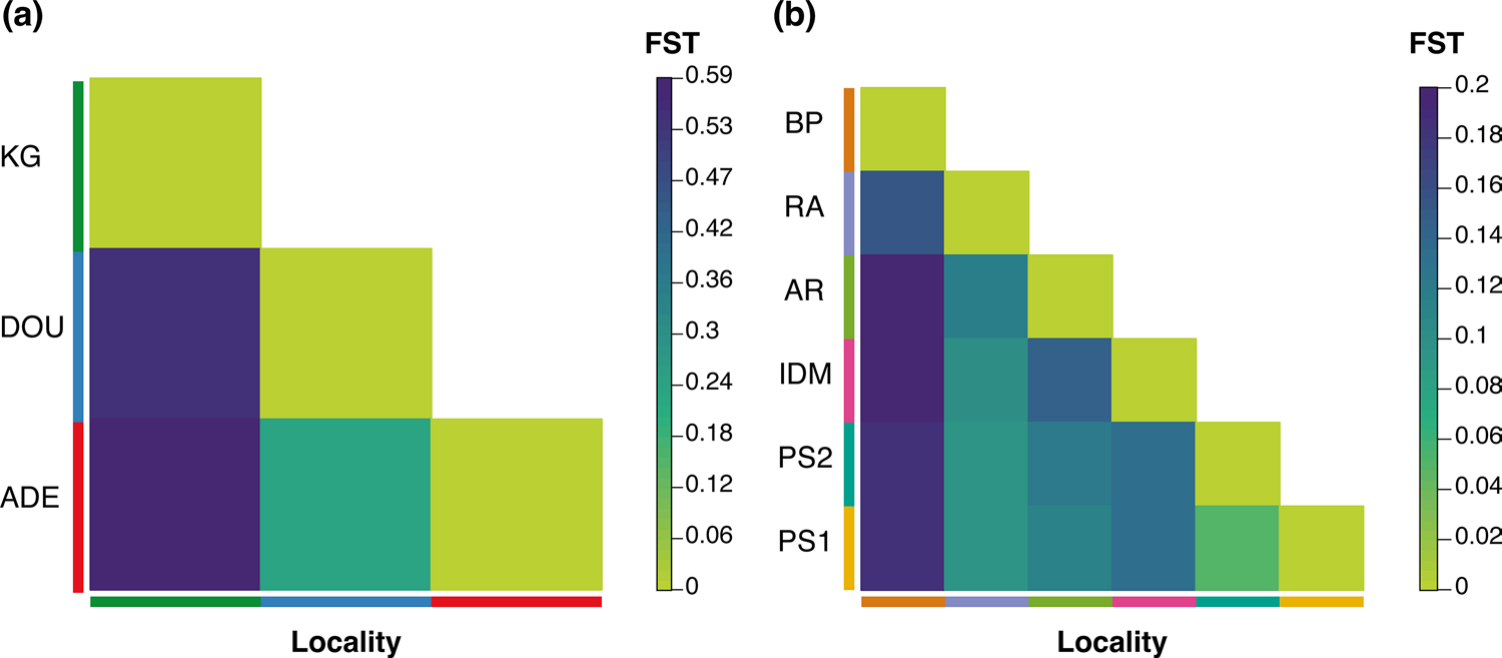
Heatmap showing pairwise FST values among locations using neutral SNPs for *Alcyonium antarcticum*. (**a**) Pairwise FST values estimated at the large sampling scale of the Antarctic Peninsula among three locations. Locations are geographically ordered from north to south; King George Island (KG), Doumer Island (DOU) and Adelaide Island (ADE). (**b**) Pairwise FST values estimated at the local/fine scale sampling scale of the Fildes Bay in King George Island among six sites. Sites in Fildes Bay correspond to: Barton Peninsula (BP), Ras Tu (RA), Artigas (AR), Islote Dos Morros (IDM), Punta Suffield rock 2 (PS2) and Punta Suffield rock 1 (PS1). Colors along the axis are the same as for the PCAs shown in Fig. 3a,b.

**Fig. 3:**
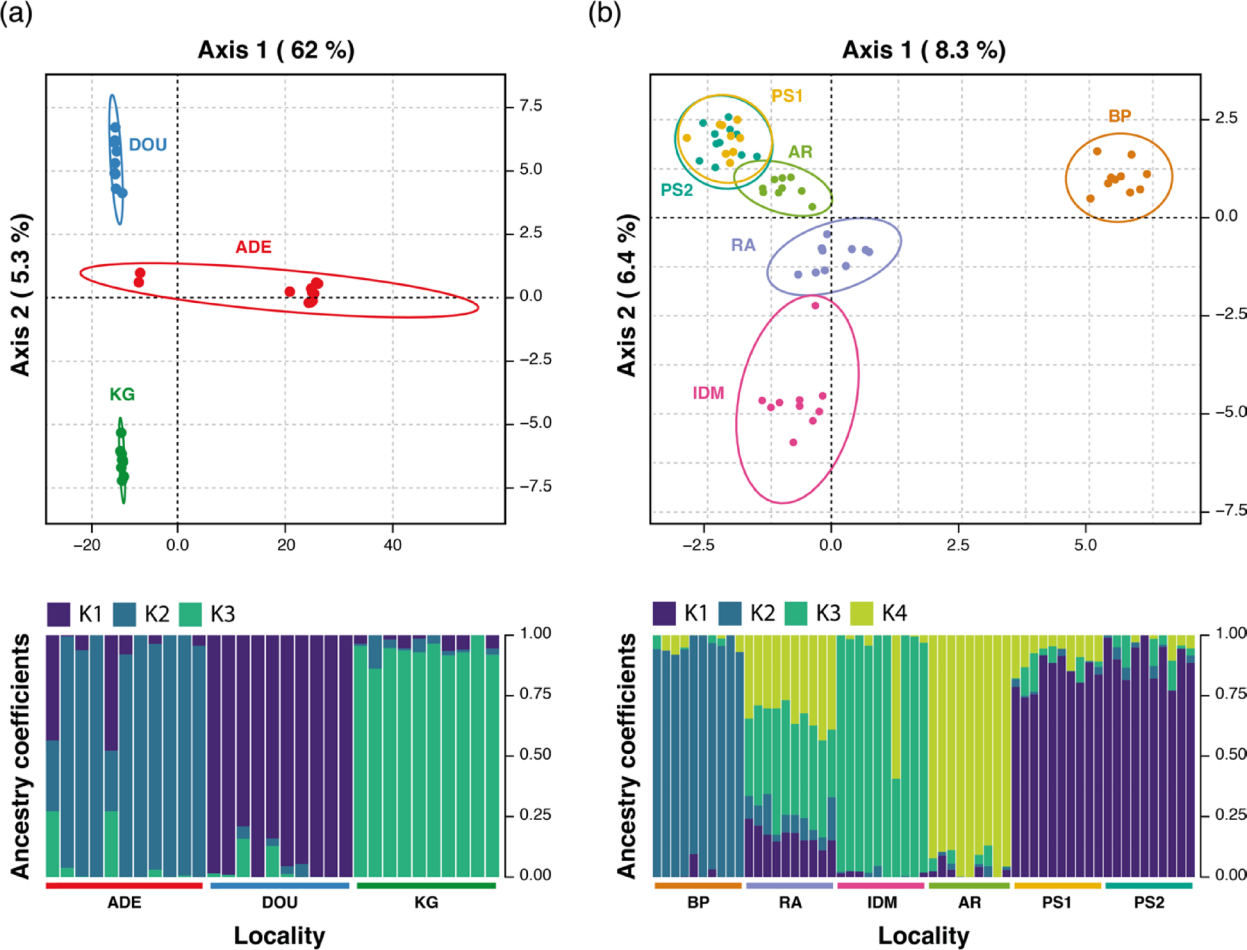
Spatial genetic structure of *Alcyonium antarcticum* at two spatial scales. Top panels: Principal coordinate analyses (PCoA) based on (**a**) large-scale sampling (1,574 SNPs), and (**b**) local/fine scale sampling (2,219 SNPs). Bottom panels: sNMF ancestry estimates for clustering at (**a**) *k* = 3 (large scale), and (**b**) *k* = 4 (local/fine scale). Colored circles in PCoA plots represent individuals from distinct localities, and vertical bars in sNMF plots indicate individual ancestry coefficients.

Pairwise FST values among ADE, DOU, and KG were high (FST = 0.259–0.600; *p* < 0.05), indicating strong genetic differentiation (Fig. 2a). No significant isolation by distance was detected at the large scale (Mantel *r* = 0.372, *p* = 0.5; Supplementary Figure S1a), although statistical power is limited because only three locations (and thus three pairwise comparisons) were available. Principal coordinate analysis (PCoA) revealed two main groups of ADE individuals along the first axis, explaining 62% of the variance, while DOU and KG were differentiated along the second axis (5.3% of variance; Fig. 3a, top). Nine ADE individuals form the cluster located on the right part of the first axis, whereas two ADE individuals occupy an intermediate position between DOU and KG. The sNMF analysis (Fig. 3a, bottom) identified three genetic clusters corresponding to ADE, DOU and KG (K = 3, lowest cross-entropy; Supplementary Figure S2a). Individuals from DOU and KG were largely assigned to their respective clusters. Two ADE individuals showed contributions from all three clusters (Q ≈ 0.25–0.40), consistent with their position in the PCoA. Given the low level of missing data (mean 2.1%), this pattern is unlikely to reflect genotyping artefacts. Clustering patterns were broadly congruent between PCoA and sNMF, with minor differences reflecting partial admixture rather than distinct lineage separation. Specifically, the two ADE individuals plotting closer to DOU/KG on the PCoA showed partial ancestry from all three clusters in the sNMF analysis (Q ≈ 0.25–0.40 per cluster), consistent with genomic admixture. K = 2 primarily captured the main ADE vs. DOU/KG split, and higher K values (up to K = 4) did not reveal additional stable genetic clusters beyond the three identified at K = 3 (Supplementary Figure S2a).

**Fig. 4:**
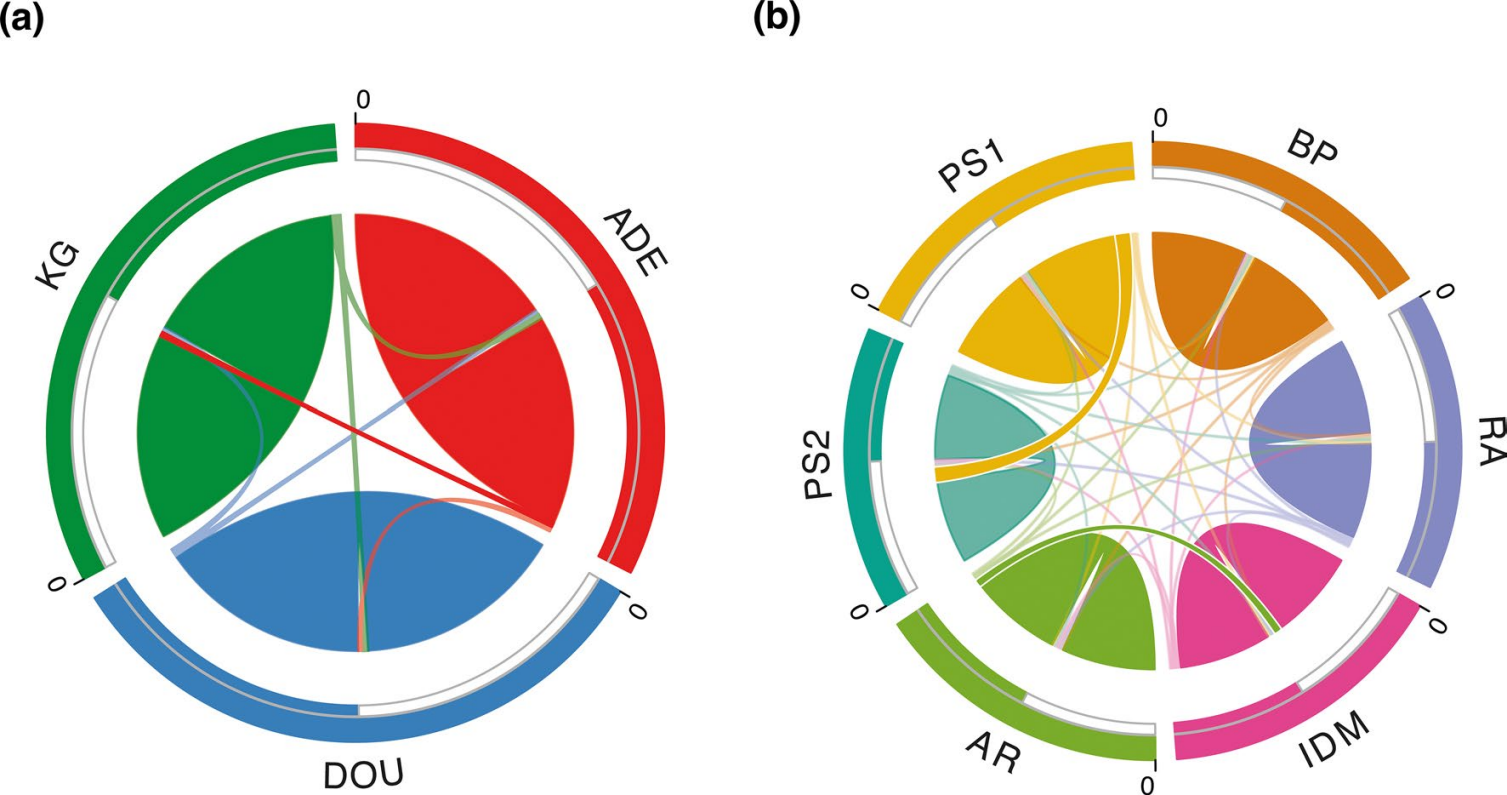
Contemporary gene flow diagrams for *Alcyonium antarcticum* as estimated with BayesAss. Contemporary gene flow estimates are represented among sampled localities at (**a**) large geographic sampling-scale and (**b**) local/fine geographic sampling-scale. Color coding is according to source locations; width of the colored arcs corresponds to the magnitude of gene flow with ‘humps’ in the estimates of gene flow representing self-assignment of individuals to their own locality (self-recruitment) and connecting arcs representing the outward gene flow from one locality to another. Patterns for each diagram are independent, that is, similar widths of arcs or humps do not represent the same amount of gene flow across the two diagrams (see Supplementary Table S1 for exact gene flow estimates). Localities are abbreviated as in Table 1.

Contemporary gene flow estimated with BayesAss indicated high self-recruitment across all populations (~ 88–90%; Fig. 4a; Supplementary Table S1). Migration among populations was generally low (< 3%). The highest migration rate was detected from ADE to KG (m = 0.029 ± 0.024); all remaining migration rates ranged between 0.020 and 0.026.

**Fig. 5:**
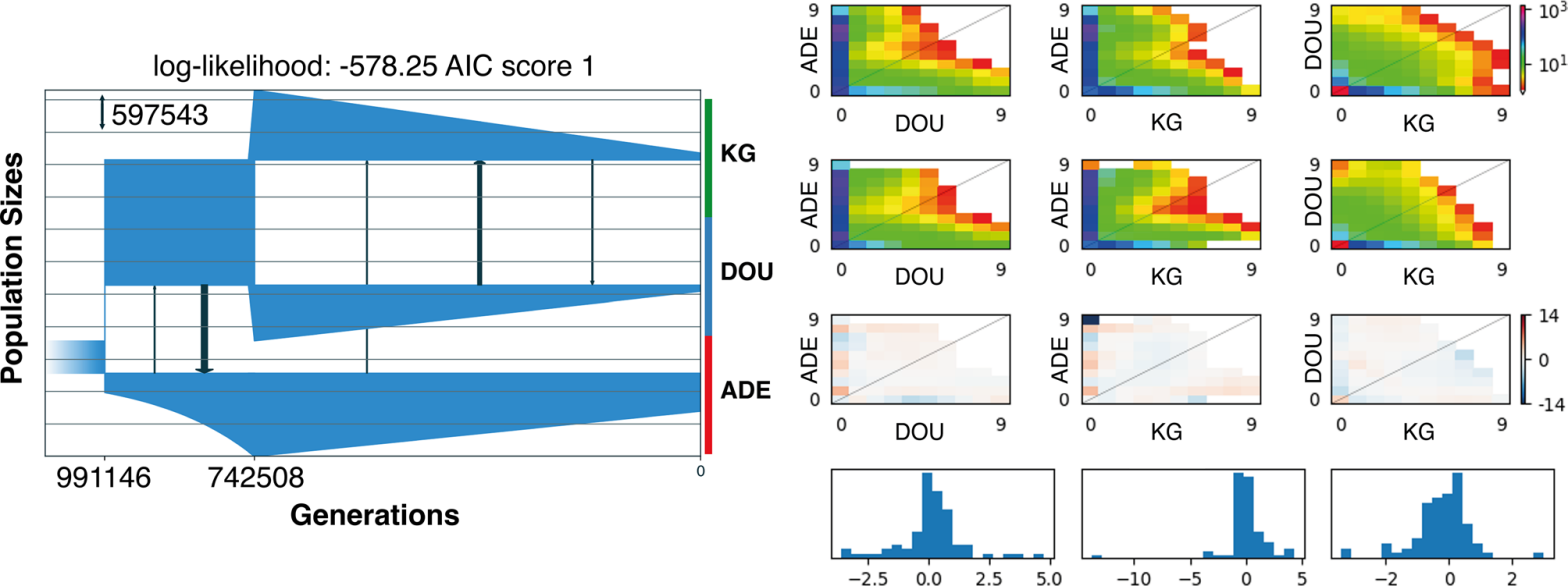
Demographic history for three main populations of *Alcyonium antarcticum.* Populations correspond to the large sampling scale of the Antarctic Peninsula; King George Island (KG), Doumer Island (DOU) and Adelaide Island (ADE). Along the x-axis, time is indicated in number of generations. Arrow with number located at the top-left indicate the scale used for population size. Allele frequency spectrum distribution of sfs files are presented on the right part of the figure (first row), theorical model sfs (second row), residuals (third row), model adjustment for each population (fourth row).

Demographic inference with GADMA (Fig. 5), based on pairwise folded 2D-SFS analyses, indicated that ADE was the first population to diverge from the ancestral lineage (~ 2 Mya), followed by divergence between DOU and KG (~ 1.5 Mya). Model fits were consistent across population pairs, supporting this sequential divergence scenario.

The ancestral population had a stable effective size (Ne) of ~ 600,000 individuals. Following divergence, all descendant populations expanded, with peak effective sizes of ~ 1.5 million individuals for ADE and of 2.3 million individuals for DOU + KG lineage. Recent population declines were detected in all three populations. Current estimates of Ne are higher for ADE (~ 700,000 individuals) than DOU (~ 112,000 individuals) and KG (~ 128,000 individuals).

### Local/fine geographic scale: genetic patterns within the Fildes Bay

No potential asexual clones were detected in the local/fine scale dataset using GENODIVE. The pairwise genetic distance distribution showed a unimodal pattern, with distances ranging from 524 to 1,119 bp (Supplementary Figure S3), indicating that all individuals can be considered as independent genotypes in subsequent analyses.

Kinship estimates based on the method of moments (MoM) and maximum likelihood estimation (MLE) approaches were strongly correlated (*r* = 0.56; Supplementary Figure S4), although they produced slightly different distributions of relatedness categories. Whatever the method used, across all populations, most individuals were inferred to be unrelated or only distantly related (i.e., first cousins at most; values of kinship coefficient ≤ 0.19). Within sites, the MoM approach consistently inferred a higher proportion of unrelated pairs (4–76%) compared to MLE (0–39%). Individuals sampled in the same site were generally unrelated, with the exception of BP, where a high proportion of pairwise relationships was inferred as half siblings (MoM: 62%; MLE: 95%). MoM also identified a few highly related individuals across sites. For example, one individual from IDM (IDM04) was inferred to be a full-sib to two individuals from IDM, three from AR, and one from BP. However, relatedness values among these pairs were low (0.00–0.08), and only the pairs BP07/BP04 and BP07/BP06 were consistently identified as full siblings.

Within the Fildes Bay, genetic diversity was relatively homogeneous among sites. Observed (H_o_) and expected (H_e_) heterozygosity values ranged from 0.088 to 0.098 and from 0.098 to 0.102, respectively, with no significant differences among sites (*p* > 0.05). Inbreeding coefficients (F_IS_) were low (0.017–0.091), except for AR (F_IS_ = 0.148); all values were statistically significant (*p* < 0.05).

Pairwise FST values among populations were moderate to high and statistically significant (FST = 0.089–0.190, Fig. 2b). The highest differentiation was found between BP and other locations (FST = 0.147–0.190). The lowest value was observed between PS1 and PS2 (FST = 0.046), which, despite being only ~ 66 m apart, showed a small but significant level of differentiation (Fig. 2b). A significant pattern of isolation by distance (IBD) was detected across the local/fine scale dataset using a Mantel test (*r* = 0.834, *p* = 0.001), indicating that geographic separation among reefs influenced genetic structure within the Fildes Bay (Supplementary Figure S1b).

In the PCoA, the first axis (explaining 8.3% of the genetic variance) clearly separates BP from all other populations (Fig. 3b, top). PS (PS1 and PS2), AR, RA and IDM are separated along the second axis (explaining 6.4% of the genetic variance). The sNMF analysis identified four genetic groups (K = 4, lowest cross-entropy; Supplementary Figure S2b). Individuals from BP, AR, IDM and PS (PS1-PS2) were predominantly assigned to distinct clusters, whereas individuals from RA showed substantial admixture among clusters. Admixture was low in BP and AR, moderate in PS, and highest in RA. In IDM, most individuals showed little admixture, except for one individual exhibiting mixed ancestry between two clusters. Overall, clustering patterns were consistent between PCoA and sNMF, indicating pronounced genetic differentiation among sites with localized admixture at RA.

Contemporary gene flow estimated with BayesAss indicated high self-recruitment across all sites (~ 77–90%; Fig. 4a; Supplementary Table S1). Migration among populations was generally low, with most pairwise estimates below 0.03. The highest inter-population migration rates were detected from AR to IDM (m = 0.042 ± 0.027) and from PS2 to PS1 (m = 0.138 ± 0.049). All remaining pairwise migration rates ranged between 0.020 and 0.023, indicating very low contemporary connectivity among most localities.

## Discussion

In this study, a multiscale sampling design combined with high‑resolution SNP genotyping was used to investigate connectivity and genetic structure in the soft coral *Alcyonium antarcticum* along the Western Antarctic Peninsula (WAP). Despite the species’ broad geographic distribution, our results reveal pronounced genetic subdivision at large scales, restricted dispersal and isolation by distance at local scales, and kin-structured neighborhoods without evidence of clones at fine scales. Together, these patterns indicate that *A. antarcticum* forms patchy, weakly connected populations whose dynamics are shaped by both historical isolation and contemporary limits to larval dispersal. Our results provide new insights into the reproductive ecology of *A. antarcticum* and how it can limit gene flow and have important implications for conservation under rapid environmental change in the WAP.

### Glacial history and oceanic barriers shape population structure along the Western Antarctic Peninsula

Our analyses revealed strong genetic differentiation of *A. antarcticum* along the WAP, with three well-defined genetic clusters corresponding to Adelaide Island (ADE), Doumer Island (DOU), and King George Island (KG). High FST values and the absence of a distance–genetic differentiation trend together suggest that geographic distance alone does not explain the observed structure. Although isolation by distance cannot be tested robustly with only three populations, the lack of a clear signal is consistent with regionally persistent constraints on connectivity along the WAP, a pattern also reported in other Antarctic marine invertebrates^46–48^.

The separation of ADE individuals along the first PCoA axis, together with previous reports of cryptic lineages in *A. antarcticum*, could suggest hidden lineage divergence. However, the same individuals analysed here were previously assigned to a single WAP lineage based on phylogeographic analyses of mitochondrial and nuclear DNA sequence markers^30^. Moreover, the two ADE individuals that plot separately show partial ancestry from all clusters in the sNMF analysis, consistent with genomic admixture rather than membership in a distinct, cohesive lineage. Taken together, these patterns are more consistent with historical structure and limited gene flow than with the presence of a distinct cryptic lineage within the WAP.

Demographic inference suggests that historical connectivity patterns along the WAP have been broadly similar to present-day ones, with populations from the three regions remaining largely isolated for hundreds of thousands of generations. The population of Adelaide Island was inferred to diverge first from the ancestral population approximately 2 Mya, followed by a split between Doumer Island and King George Island around 1.5 Mya. These timings broadly coincide with the Mid-Pleistocene Transition, a period characterized by intensified glacial cycles and major changes in Antarctic ice extent and sea level^49,50^. We propose that glacially driven cycles of habitat contraction and isolation promoted the divergence of *A. antarcticum*along the WAP. Similar processes are well known to have driven divergence and diversification of marine invertebrates at high latitudes in both the Southern Hemisphere (e.g.,^51^) and the Northern Hemisphere (e.g.,^52^).

Expected heterozygosity in *Alcyonium antarcticum* (H_e_: 0.076–0.247; Table 1) was consistently low compared with values reported for temperate and tropical octocorals analyzed using microsatellite loci, where mean H_e_ ranges from 0.42 in *Eunicella verrucosa* to 0.74 in *Corallium rubrum* and *Paramuricea clavata *(see Holland, et al.^10^ and references therein). However, this comparison should be interpreted cautiously, as SNPs and microsatellites differ fundamentally in mutation rate, allele number, and ascertainment bias, making direct comparisons of heterozygosity across marker types unreliable^53,54^. Estimates of genetic diversity based on SNP datasets in corals and octocorals are generally within a similar range to those observed here^55,56^. Nonetheless, the relatively low genetic diversity in *A. antarcticum* is consistent with patterns reported for several Antarctic marine taxa and may partly reflect the legacy of repeated population bottlenecks during glacial periods, when ice-sheet advances reduced and fragmented suitable shelf habitats^50^.

Our interpretations of genetic diversity in *A. antarcticum* are necessarily framed within the Neutral Theory framework^57^, which, while useful as a null model, has faced increasing empirical challenge. Evidence from comparative genomics and functional analyses indicates that molecular patterns previously assumed to be neutral can arise from selective or adaptive processes^58–60^. Accordingly, while we interpret the reduced genetic diversity observed in *A. antarcticum* as consistent with glacial bottlenecks (see above), selection-linked genomic architecture may also contribute. Our conclusions regarding genetic diversity should therefore be treated as comparative and model-dependent, and future work integrating whole-genome sequencing with functional annotation and selection scans will be needed to fully disentangle the contributions of neutral demographic processes from selective forces shaping diversity in this species.

Despite the generally low levels of genetic diversity detected in *A. antarcticum*, we observed higher heterozygosity in the Adelaide Island population compared with those from Doumer Island and King George Island. This pattern may reflect the historical role of Adelaide Island as a glacial refuge during successive glacial cycles. Bayesian demographic inferences conducted using GADMA indicate that populations across all three regions began contracting following the second genetic divergence event (~ 1.5 Ma; Fig. 5), although population declines were substantially more pronounced at Doumer Island and King George Island than at Adelaide Island. Similar recent population reductions have been documented in other Antarctic benthic invertebrates^61–63^. While glacial impacts might be expected to intensify toward lower latitudes in the southern portion of the WAP, extensive glacial refugia have been reported in the Adelaide Island region^64,65^. Local ice dynamics in this region may therefore have facilitated the persistence of relatively large *A. antarcticum* populations throughout glacial periods.

Other studies have detected genetic isolation between populations located in the regions sampled in the present study. A subtle but consistent genetic structure similar to that observed in *A. antarcticum* has also been detected in other Antarctic marine organisms using SNP data, including one fish (*Harpagifer antarcticus*^66^, two sponges (*Mycale acerate* and *Dendrilla antarctica*^67,68^ and two nemertean worms (*Antarctonemertes valida* and *A. riesgoae*^68^), as well as in terrestrial invertebrates such as midges and springtails^65,69^. However, in all these other organisms, gene flow was generally high, likely reflecting greater dispersal potential. In contrast, *A. antarcticum* could have a more restricted dispersal associated with its reproductive mode. We propose that the species could be a brooder releasing lecithotrophic planula larvae, as most of the *Alcyonium* species for which the reproductive mode has been determined (see below for more detail), a characteristic that could limit dispersal distances and may promote local larval retention, thereby reducing gene flow^10,16,70,71^.

Interestingly, two individuals with highly admixed genetic ancestries were detected at Adelaide Island, indicating a low amount of contemporary gene flow from both Doumer Island and King George Island toward Adelaide Island. The Antarctic Peninsula Coastal Current, which is strongly influenced by the southward outflow from the Weddell Sea and flows southward along the Western Antarctic Peninsula^72^, could facilitate larval transport from Doumer Island to Adelaide Island. In contrast, the Antarctic Coastal Current flowing northward along the outer shelf of the WAP bifurcates upon reaching the southern South Shetland Islands: the main branch continues along the outer coasts of the South Shetland Islands, while a narrower branch—the Bransfield Current—flows along the inner slope, forming the Bransfield Front^73^. This frontal system may act as a partial barrier to larval exchange between King George Island and Adelaide Island. Nevertheless, strong anticyclonic eddies reported in this region could intermittently weaken the Bransfield Front, thereby enabling occasional larval exchange across this boundary^73^.

### Fine-scale population structure, reproductive mode, dispersal limitation, and local retention in Fildes Bay

Across all localities sampled within Fildes Bay (King George Island), no repeated multilocus genotypes were detected, and inbreeding coefficients were consistently low (Table 1; Supplementary Fig. 3), indicating that *A. antarcticum* reproduces predominantly through sexual reproduction with cross-fertilization among unrelated colonies. This pattern was maintained even at the finest spatial scale, as colonies separated by only a few centimeters on the same rock were genetically distinct. These results rule out clonality as a driver of local genetic structure and contrast with congeneric species such as *Alcyonium rudyi*, in which binary fission produces spatially aggregated clonal patches^74^.

Despite the predominance of sexual reproduction, genetic differentiation among reefs was pronounced at small spatial scales. Pairwise *F*ST values among sites separated by only a few kilometers were moderate to high and statistically significant (Fig. 2), and a strong isolation-by-distance (IBD) signal was detected within Fildes Bay. Multivariate and Bayesian clustering analyses further resolved discrete genetic groups largely corresponding to individual sites, with limited admixture and high levels of inferred self-recruitment (Figs. 3 and 4). Together, these patterns indicate that connectivity declines sharply over short distances and support a metapopulation framework in which subpopulations are primarily maintained by local recruitment, with only limited exchange of migrants. Such spatially restricted connectivity is consistent with patterns reported in temperate octocorals^10^ and in species exhibiting partial self-recruitment, including *Seriatopora hystrix*^75^ and *Corallium rubrum*^76^. Kinship analyses provided independent support for restricted dispersal at local scales. Only a small number of half-sibling and occasional full-sibling pairs were detected among localities across Fildes Bay, consistent with predominantly localized recruitment. Within sites, the results of kinship analyses were more variable with the Barton Peninsula (BP) locality exhibiting a higher frequency of related individuals, with a predominance of half-sibling relationships, than all other populations. This pattern could suggest the occurrence of collective dispersal, whereby cohorts of related larvae are released and settle in close spatial and temporal proximity^77^.

Species of the genus *Alcyonium* exhibit diverse reproductive strategies, including broadcast spawning of gametes into the plankton, internal brooding of embryos to varying stages of larval development, and external brooding^71^. Among the 12 *Alcyonium* species examined by McFadden et al. (2001) for which reproductive information was available, the majority were brooders (9 of 12 species; 75%). Taken together, our local- and fine-scale genetic results support the hypothesis that *A. antarcticum* is a brooding species rather than a broadcast spawner, releasing larvae with very limited dispersal capacity, likely lecithotrophic planulae. This interpretation is further supported by observations in *Alcyonium haddoni*, a recently diverged sister species restricted to the cold coastal waters of Chile, in which larvae have been observed through the transparent body wall of polyps, consistent with brooding^78^. The pronounced genetic structure detected over spatial scales of only a few kilometers in *A. antarcticum* is also consistent with patterns reported for other brooding coral species^10,75,79^. Moreover, studies of Antarctic octocorals such as *Thouarella variabilis*, *Fannyella rossii*, and *F. spinosa* indicate that seasonal reproduction and brooding are common strategies in cold environments and are often associated with elevated genetic differentiation among isolated populations^80–82^. Brooding is widespread among Antarctic invertebrates, including bivalves, gastropods, sea stars, and octocorals, and is widely regarded as an adaptation to low temperatures and limited food availability that constrain the survival of free-swimming larvae. By providing nourishment and protection during early development, brooding enhances offspring survival but typically results in reduced dispersal potential^81–83^.

### Implications for conservation under rapid warming

Despite the low genetic diversity detected in *A. antarcticum* within our study area, the combination of exclusive sexual reproduction and high self-recruitment at fine spatial scales may help maintain local adaptive potential, provided effective population sizes remain above critical thresholds. However, strong spatial genetic structure and limited connectivity among regions suggest that losses of local populations are unlikely to be rapidly compensated by immigration, potentially constraining species-wide adaptive capacity under ongoing climate change.

The WAP is among the fastest-warming regions globally, and benthic organisms are increasingly exposed to rising temperatures^37,38^, intensified iceberg scouring, ocean acidification, and altered coastal circulation^39,40^. In this context, the genetic structure documented here challenges the assumption—often implicit in regional conservation planning—that connectivity among sites is sufficient to ensure recolonization and demographic rescue following local disturbances^3,10,11,84^. Instead, our results indicate that many *A. antarcticum* populations function as semi-independent demographic and evolutionary units shaped by restricted dispersal and strong local retention.

Recent advances in comparative phylogeography and comparative population genomics in the Southern Ocean demonstrate that community-level barriers and fine-scale genetic structure are often only detectable using genome-wide markers, and that such information can be translated into map-based priorities directly relevant for MPA design^8,67,68,85^. Our SNP-based results provide this level of resolution for a habitat-forming octocoral in the WAP and indicate that integrating genomic connectivity metrics into MPA planning frameworks is essential to avoid overestimating functional connectivity.

Finally, recent policy analyses emphasize that progress on Southern Ocean MPAs is limited not only by geopolitical constraints, but also by scientific and managerial uncertainty—particularly regarding baseline data and the implementation of Research and Monitoring Plans (RMPs) under the Commission for the Conservation of Antarctic Marine Living Resources (i.e., CCAMLR^86^). Integrating genomic indicators of connectivity, demographic independence, and genetic diversity into RMPs would provide transparent, measurable benchmarks for evaluating MPA performance and may reduce recurrent objections related to data sufficiency and uncertainty in ongoing MPA negotiations.

Overall, for species such as *A. antarcticum*, conservation success in the WAP will depend less on the mere designation of MPAs than on how well protected areas reflect the true spatial scales of connectivity, demographic independence, and long-term evolutionary resilience in a rapidly changing Antarctic coastal seascape.

## Methods

### Sample collection

Samples of *Alcyonium antarcticum* were collected in 2020 along the Western Antarctic Peninsula (WAP) (Table 1) under scientific permit N°147/2020 issued by the Chilean Antarctic Institute (INACH). At the large spatial scale (hundreds of kilometers), three locations separated by 350–750 km—King George Island (KG), Doumer Island (DOU), and Adelaide Island (ADE)—were sampled (Fig. 1a). At the local scale (a few kilometers), five locations were sampled on King George Island: Punta Suffield (PS), Islote Dos Morros (IDM), Artigas (AR), Ras Tu (RA), and Barton Peninsula (BP) (Fig. 1b). The distances between locations ranged from 2.43 km (between Punta Suffield and Artigas) to 8.88 km (between Punta Suffield and Barton Peninsula). For the fine scale (within tens of meters), two patches of *A. antarcticum* colonies from Punta Suffield were sampled on separate vertical rocky walls (reefs)—Punta Suffield rock 1 (PS1) and Punta Suffield rock 2 (PS2)—approximately 66 m apart; both patches were fully sampled (Fig. 1c). All samples were obtained by SCUBA diving at a depth of 10–15 m. Tissue of *A. antarcticum* (~ 2 cm³) was collected, preserved in 96% ethanol, and stored at 4 °C. The total number of individuals and the number collected per locality are reported in Table 1.

### DNA extraction, SNP library preparation, genotyping and filtering

DNA was isolated from 20 to 30 mg of tissue using the E.Z.N.A.^®^ Tissue DNA Kit (Omega Bio-Tek Inc., USA), according to the manufacturer’s protocol. DNA integrity was assessed visually on 1.0% agarose gels and DNA concentration (ng/µL) was measured on a spectrophotometer (Nanodrop 1000, Thermo Scientific). Genomic DNA yield was also estimated, using a fluorimeter and fluorescent DNA-binding dye (Qubit™ dsDNA BR Assay Kit; Thermo Fisher Scientific), according to the manufacturer´s instructions. Genomic DNA extracts were standardized to a concentration of 50 ng/µL and sent to Diversity Arrays Technology (DArT Pty Ltd., Australia) for DArTseq™ library preparation and genotyping^87^. Reduced representation libraries were generated using Pstl-HpaII restriction enzymes and sequenced on an Illumina HiSeq 2500 platform. The raw reads were processed by DArT’s variant calling pipeline to identify Single Nucleotide Polymorphisms (SNPs).

SNPs data were filtered using the dartR V.1.1.11 package^88^ in R v3.6.0 (R Core Team, 2019) using the RStudio interface (RStudio Team, 2018). The filtering was performed according to the following criteria: loci with repeatability < 0.99, call rate < 0.95, and minor allele frequency < 0.05 were removed. To minimize physical linkage, only one SNP was retained per sequence fragment using the ‘best’ method in the gl.filter.secondaries function. Loci showing significant departures from Hardy–Weinberg equilibrium (HWE), assessed at α = 0.05 with Bonferroni correction, were also discarded. To ensure that downstream analyses were based solely on neutral genetic variation, we screened all loci for signals of selection using two independent methods: BayeScan V.2.1^89^ and OutFLANK V.0.2^90^, both implemented in dartR package with default parameters and a false discovery rate (FDR) of 0.05. Only putatively neutral SNPs retained by both methods were used in subsequent analyses.

Based on the filtering pipeline described above, two datasets were generated to match the hierarchical spatial design of the study. The large-scale dataset (*n* = 31) included individuals from KG, DOU, and ADE (Table 1; Fig. 1). All samples representing the KG region were sourced exclusively from the BP site. The local/fine-scale dataset (*n* = 59) included individuals from IDM, AR, RA, BP, PS1, and PS2 (Table 1; Fig. 1). This dataset explicitly incorporated fine-scale sampling sites (PS1 and PS2, Fig. 1) to assess genetic structure, kinship, and recruitment dynamics among colonies separated by only tens of meters. For analytical purposes, PS1 and PS2 were treated as independent reef units in local scale analyses, allowing the evaluation of genetic differentiation across very short spatial distances.

### Data analysis

Analyses were conducted according to spatial scale. Demographic history was assessed exclusively at the large spatial scale. Genetic diversity and population structure were evaluated across all spatial scales, while clonality and kinship were examined only at the local and fine scales. All analyses were conducted using the datasets described above, in accordance with the scale studied.

#### Large scale analyses: genetic diversity, population structure, admixture, gene flow, and demographic inference

Genetic diversity indices were calculated using the divBasic function in the diveRsity V.1.9.90 package^91^. For each location, we estimated observed heterozygosity (H_o_), expected heterozygosity (H_e_), and inbreeding coefficient (F_IS_). The 95% confidence intervals for F_IS_ were obtained via 1,000 bootstrap replicates. Statistical differences in Ho and He among populations were inferred by comparing locus-based bootstrap distributions (1,000 replicates) generated in diveRsity V.1.9.90. Pairwise differences were considered statistically significant when 95% confidence intervals did not overlap.

Genetic differentiation among regions was assessed using pairwise FST estimates calculated with the StAMPP package v1.5.1, with statistical significance evaluated through 1,000 bootstrap replicates. Isolation by distance (IBD) was assessed using Mantel tests correlating genetic differentiation, expressed as FST/(1 − FST), with the natural logarithm of geographic distances (km). Mantel tests were implemented using the sp package in R^92^, and statistical significance was assessed using 10,000 permutations.

Population structure was further explored using principal coordinates analysis (PCoA) implemented in dartR v1.1.11^88^. The number of informative axes retained for visualization was determined by inspecting scree plots of eigenvalues^93^. Population admixture was evaluated using the LEA package v2.6^94^. The optimal number of ancestral clusters (K) was determined with the snmf function based on the cross-entropy criterion. Although cross-entropy was used to identify the optimal K, alternative K values were also inspected to ensure biological consistency of clustering patterns. Contemporary migration rates (Nm) were estimated using BayesAss v3.0.4^95^. Three independent runs were conducted with 10⁷ iterations, a burn-in of 10⁶ iterations, and a sampling interval of 1,000. Mixing parameters (DeltaA, DeltaF, DeltaM) were adjusted to optimize acceptance rates and set to 0.8, 0.019, and 0.35. Markov chain Monte Carlo (MCMC) convergence was assessed using Tracer v1.7.1^96^. Migration patterns were visualized using circlize v0.4.12 and tidyverse v1.3.0^97^.

Historical divergence and demographic parameters were inferred using GADMA (Genetic Algorithm for Demographic Model Analysis)^98^. Joint site frequency spectra were generated as folded two‑dimensional SFS (2D‑SFS) for each population pair using the easySFS Python script, given the absence of a suitable outgroup. For each pair, we compared alternative demographic models representing different divergence scenarios (including changes in effective population size and asymmetric migration) and retained the best‑supported model based on likelihood and information‑criterion scores. Multiple independent GADMA runs (*n* = 30 per model) were performed per model to assess convergence, and the robustness of model choice and parameter estimates was evaluated by comparing results across runs and population pairs.

Demographic models represented sequential divergence of three populations from an ancestral lineage (two split events), including alternative migration scenarios (no migration, symmetric migration, and asymmetric migration after divergence) with effective population sizes allowed to vary among lineages. Model parameters included divergence times, effective population sizes, and migration rates. Model optimization was performed using the *moments* engine^99^, selected for its robustness and computational efficiency.

For each population pair, 10 replicate optimizations were performed per model, and the best-fitting model was selected based on log-likelihood and Akaike Information Criterion (AIC). Consistency of model support and parameter estimates was evaluated across the three pairwise comparisons (ADE–DOU, ADE–KG, DOU–KG) to assess robustness of demographic inference. Parameter scaling used a mutation rate of 1.38 × 10^− 9^ site/year, as reported for corals^100^ and a generation time of two years. In the absence of species-specific estimates for *Alcyonium antarcticum*, generation time was inferred from closely related soft coral taxa, for which values of approximately 1–3 years have been reported for octocorals, including *Alcyonium digitatum*^71,101^, *Alcyonium coralloides*^102^ and *Alcyonium acaule*^103^. Accordingly, we adopted a conservative generation time of two years for *A. antarcticum*, consistent with maturation ages documented for octocorals^104^.

#### Local and fine scale analyses: genetic diversity, population structure, admixture, gene flow, clonality, and relatedness

Genetic diversity, population structure, and genetic differentiation at the local and fine spatial scales were assessed using the same approaches as described for the large-scale analyses (Sect. 2.3.1). In addition, we further examined clonality and kinship at the local and fine spatial scales.

To detect potential clonal individuals, we used GENODIVE v3.06 under the infinite-allele model (IAM), which is appropriate for biallelic SNP data, excluding missing genotypes. To assess kinship at neighborhood level (local/fine scales), kinship coefficients were estimated using two approaches: the method of moments (MoM)^105^ and a maximum likelihood estimator (MLE)^106^, both implemented in SNPRelate V.1.18.0^107^. For both methods, we followed the default instructions provided in the SNPRelate package vignette (https://bioconductor.org/packages/release/bioc/vignettes/SNPRelate/inst/doc/SNPRelate.html). The estimated degree of relatedness ranges from 0 (unrelated) to 0.50 (self-fertilization). Pairs of samples with kinship coefficient (ϕ) ranging from 0.00 to ≤ 0.03 were considered unrelated, from 0.03 to ≤ 0.09 as first cousins, from 0.09 to ≤ 0.19 as half-sibs and from 0.19 to 0.25 as full-sibs.

## Supplementary Information

Below is the link to the electronic supplementary material.

Supplementary Material 1

## Data Availability

The SNP datasets generated using DArTseq are available in Zenodo (https://doi.org/10.5281/zenodo.18174803).
