# Supplementary material for: Multiscale genetic connectivity, kinship, and demographic history of the Antarctic soft coral Alcyonium antarcticum in the Western Antarctic Peninsula
Source: Sci Rep. 2026 Apr 26;16:19729. doi: 10.1038/s41598-026-47577-5 (PMC13316029; doi:10.1038/s41598-026-47577-5)
Supplement: Supplementary file 1 — Supplementary Material 1 [file 41598_2026_47577_MOESM1_ESM.docx]

SUPPLEMENTARY FILE


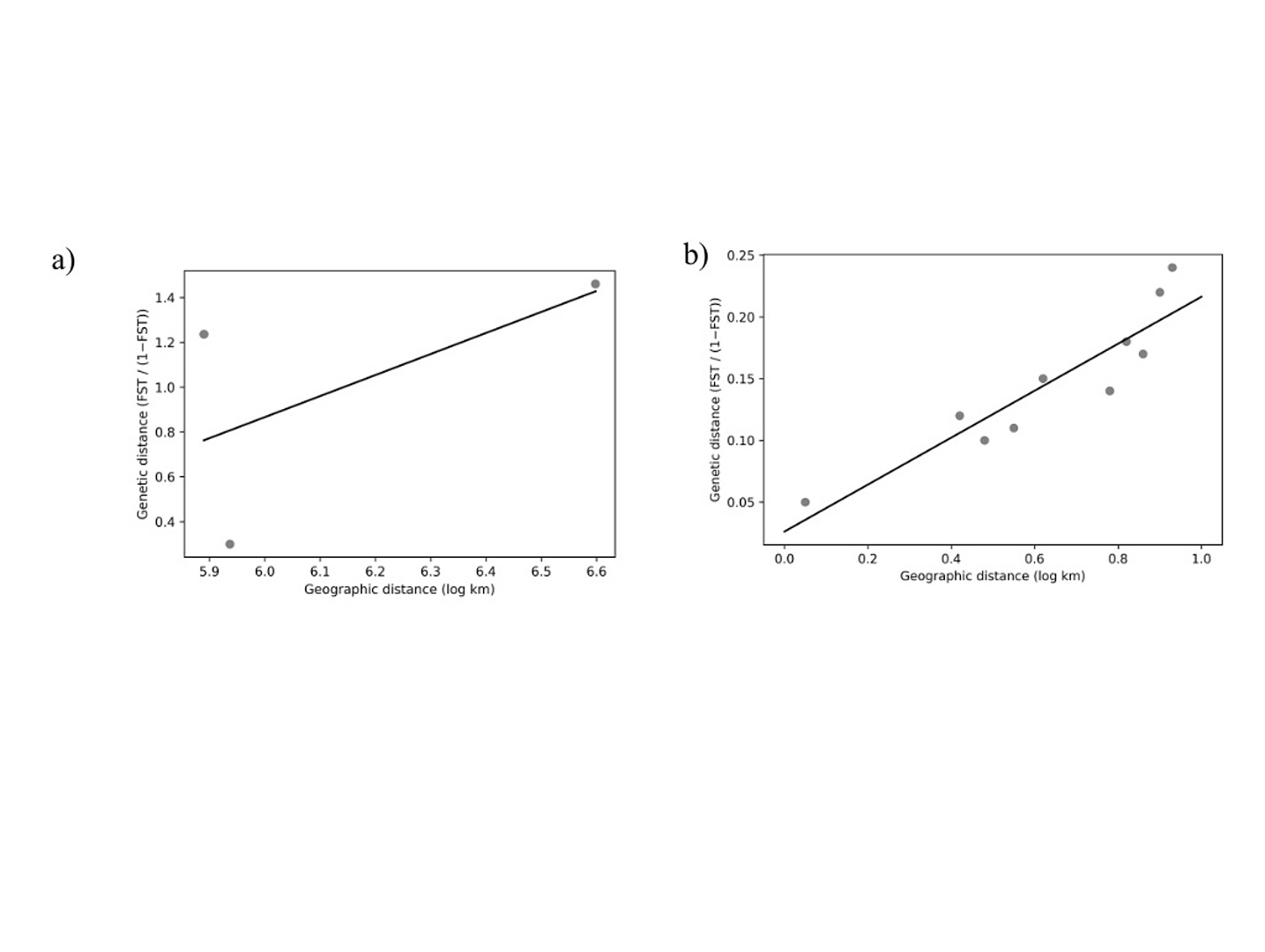


**Figure S1.** Isolation-by-distance plot of *F*_ST_/1−*F*_ST_ versus population geographical distance among populations. The results are shown for (a) large scale (i.e., along the Western Antarctic Peninsula) and (b) local scale analyses (i.e., within the Fildes Bay, King George Island).

**Figure S2.** Cross-entropy values across tested numbers of genetic clusters (K), shown for (a) large scale and (b) local scale analyses.


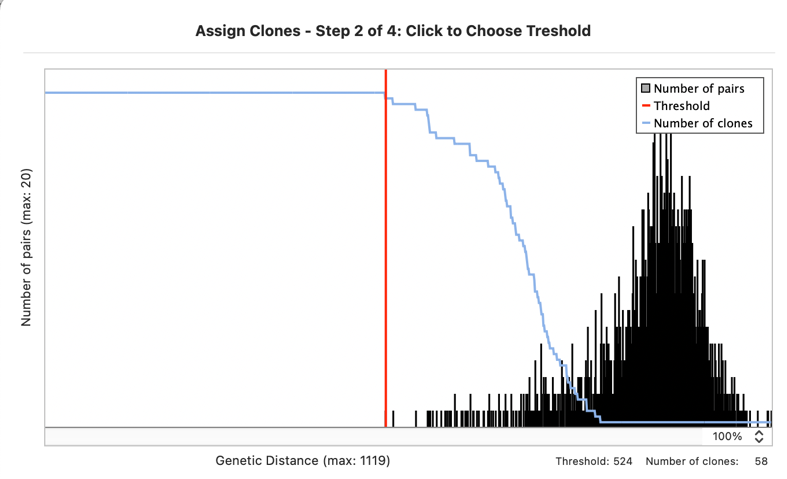


Figure S3: AssignClones plot illustrating the relationship between pairwise genetic distances and the number of genetically identical multilocus genotypes in the octocoral *Alcyonium antarcticum.* Black bars represent the frequency distribution of pairwise genetic distances, while the blue line indicates the cumulative number of inferred clones across increasing distance thresholds. The vertical red line marks the selected genetic distance threshold used to define clonal individuals. This threshold was chosen at the point where the number of inferred clones stabilizes, minimizing the risk of misclassifying closely related but sexually produced individuals as clones.


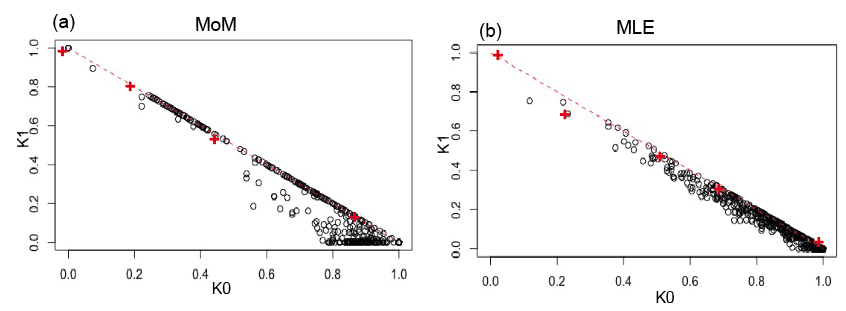


**Figure S4.** Pairwise IBD coefficients (k0 versus k1) estimated in PLINK using (a) the method of moments (MoM) and (b) the maximum likelihood estimation (MLE). Red crosses indicate theoretical values for Parent-Offspring (k0 = 0, k1 = 1), Full Siblings (k0 = 0.25, k1 = 0.5), Half Siblings (k0 = 0.5, k1 = 0.5), **First Cousins** (k0 = 0.75, k1 = 0.25) and **Unrelated** (k0 = 1, k1 = 0) relationships.

**Table S1.** Matrix of inferred contemporary migration rates (m) for Alcyonium antarcticum estimated using BayesAss v3.0.4. Values correspond to posterior means with standard deviations in parentheses, summarized across independent runs. Rows represent the **recipient population (To)** and columns the **source population (From)**. Diagonal values (in **bold**) indicate **self-recruitment**.

(a) Large spatial scale

|  | **ADE** | **DOU** | **KG** |
| --- | --- | --- | --- |
| **ADE** | **0.8961 ± 0.0345** | 0.0209 ± 0.0256 | 0.0209 ± 0.0256 |
| **DOU** | 0.0207 ± 0.0237 | **0.8961 ± 0.0321** | 0.0208 ± 0.0238 |
| **KG** | 0.0294 ± 0.0237 | **0.0208 ± 0.0322** | **0.875 ± 0.0375** |

(b) Local / fine spatial scale

|  | **BP** | **RA** | **IDM** | **AR** | **PS1** | **PS2** |  |
| --- | --- | --- | --- | --- | --- | --- | --- |
| **BP** | **0.8955 ± 0.038** | 0.0211 ± 0.010 | 0.0208 ± 0.019 | 0.0211 ± 0.020 | 0.0207 ± 0.020 | 0.0208 ± 0.020 | |
| **RA** | 0.0209 ± 0.020 | **0.8952 ± 0.038** | 0.0211 ± 0.020 | 0.0209 ± 0.020 | 0.0211 ± 0.020 | 0.0208 ± 0.020 | |
| **IDM** | 0.0208 ± 0.020 | 0.0206 ± 0.020 | **0.8746 ± 0.040** | 0.0421 ± 0.027 | 0.0209 ± 0.020 | 0.0210 ± 0.020 | |
| **AR** | 0.0223 ± 0.021 | 0.0224 ± 0.020 | 0.0219 ± 0.021 | **0.8892 ± 0.040** | 0.0220 ± 0.021 | 0.0221 ± 0.021 | |
| **PS1** | 0.0222 ± 0.021 | 0.0222 ± 0.021 | 0.0223 ± 0.021 | 0.0222 ± 0.021 | **0.7733 ± 0.048** | 0.1378 ± 0.050 | |
| **PS2** | 0.0199 ± 0.019 | 0.0196 ± 0.019 | 0.0196 ± 0.018 | 0.0196 ± 0.018 | 0.0196 ± 0.018 | **0.9017 ± 0.036** | |
